# Towards the Development of a Strategy to Characterize and Model the Rheological Behavior of Filled, Uncured Rubber Compounds

**DOI:** 10.3390/polym13234068

**Published:** 2021-11-23

**Authors:** M. M. A. Spanjaards, G. W. M. Peters, M. A. Hulsen, P. D. Anderson

**Affiliations:** 1 Department of Mechanical Engineering, Eindhoven University of Technology, P.O. Box 513, 5600 MB Eindhoven, The Netherlands; m.m.a.spanjaards@tue.nl (M.M.A.S.); g.w.m.peters@tue.nl (G.W.M.P.); m.a.hulsen@tue.nl (M.A.H.); 2 VMI Holland B.V., Gelriaweg 16, 8161 RK Epe, The Netherlands

**Keywords:** fillers, rubber compounds, viscoelasticity, thixotropy, structure

## Abstract

In this paper, an experimental strategy is presented to characterize the rheological behavior of filled, uncured rubber compounds. Oscillatory shear experiments on a regular plate-plate rheometer are combined with a phenomenological thixotropy model to obtain model parameters that can be used to describe the steady shear behavior. We compare rate- and stress-controlled kinetic equations for a structure parameter that determines the deformation history-dependent spectrum and, thus, the dynamic thixotropic behavior of the material. We keep the models as simple as possible and the characterization straightforward to maximize applicability. The model can be implemented in a finite element framework as a tool to simulate realistic rubber processing. This will be the topic of another work, currently under preparation. In shaping processes, such as rubber- and polymer extrusion, with realistic processing conditions, the range of shear rates is far outside the range obtained during rheological characterization. Based on some motivated choices, we will present an approach to extend this range.

## 1. Introduction

Rubber extrusion is widely used in the automotive industry to produce, for example, rectangular rubber strips, which can be stripwinded into the carcass of a car tire, and weather strips that seal a car door and prevent rainwater and dust from coming in. Rubber-like materials are typically viscoelastic and show nonlinear and time-dependent rheological behavior under stress. The dimensions and quality of the rubber extrusion products highly depend on the rheological properties of the compound. Rubber compounds are complex materials in the sense that they contain many additives, such as plasticizers, curing agents, and they contain about 30% by weight of reinforcing fillers [1].

Carbon black and silica are the two most popular types of fillers in the automotive industry to improve the mechanical behavior of rubber products. They are both active fillers, which means they interact with the polymer matrix. The reinforcing effect of the fillers is therefore based on polymer–filler, as well as filler–filler interactions. Since carbon-black suspensions are used in numerous industrial applications, ranging from tire rubbers to ink and electrochemical energy storage devices [2,3,4], they are considered as highly relevant thixotropic colloidal suspensions. Therefore, this paper will focus on carbon-black reinforced rubber compounds.

**Figure 1:**
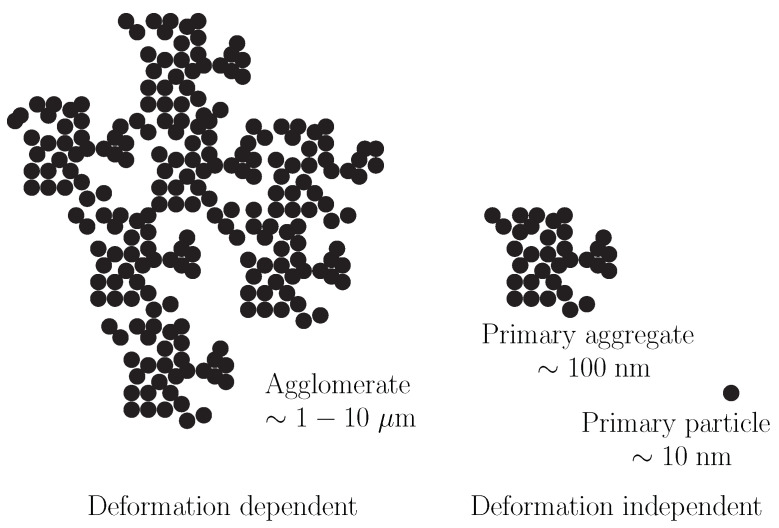
Schematic representation of carbon-black aggregates and the flocculation into agglomerates.

The dispersion of the fillers in the polymer matrix is critical; the better the dispersion, the better the performance properties of the compound [5,6]. Furthermore, the addition of carbon-black increases the viscosity which leads to a change in the flow characteristics of the compound. The primary carbon-black particles form aggregates and the size and shape of these aggregates are deformation-independent. These aggregates can cluster together to form agglomerates, which can form a network that is held together by weak van der Waals-type forces. These networks are very sensitive to even small changes in strain and continue to separate as the strain increases [7]. These different types of carbon-black structures are schematically depicted in Figure 1. The fragile bonds between the agglomerates can break under stress but will reform again once the stress is removed. This leads to a reversible decrease in the viscosity. This thixotropic behavior is also known as the Payne effect, and makes rheological characterization of the compounds challenging [8]. When shear is applied to carbon-black suspensions, shear-thinning behavior is observed which is consistent with structural break-down of these agglomerates [9,10,11].

The Payne effect has been attributed to several mechanisms, such as the agglomeration/de-agglomeration of filler aggregates, breakup/reformation of the filler–filler and polymer–filler network [12,13], chain desorption from the fillers [14], yielding of the glassy layer between the fillers [15] and disentanglement of the absorbed chains [16].

Considerable research has been performed to characterize the dynamic mechanical properties of filled rubber compounds [17,18,19,20,21,22,23,24]. Addition of fillers leads to two contributions to the dynamic modulus of the material; a strain-dependent contribution and a strain-independent one. The modulus decreases with increasing strain due to a partial reversible breakdown of the filler–filler network, whereas filler–rubber interactions contribute to the strain-independent part [25]. The reversible networks created by the filler particles also result in additional elastic behavior of the compound.

The concept of thixotropy was introduced by Péterfi [26]. Since then, the modeling of thixotropy has been studied extensively and is considered as one of the most challenging problems in colloid rheology. The underlying changes in microstructure are often complex and still poorly understood. In the review of Larson and Wei [27], a summary is given of the most used models to describe thixotropic behavior. In their review, they restrict the term thixotropy to nearly inelastic behavior so that it cannot be confused with non-linear viscoelasticity. Mewis and Wagner described a definition of thixotropy that is based on viscosity [9]. This definition implies a time-dependent decrease of viscosity induced by flow that is reversible when the flow is decreased or arrested.

Most simplistic thixotropy models are based on a kinetic equation that describes the evolution of a structure parameter to express the state of the structure in the material. A distinction is made between rate- and stress-controlled models. The basic form of a rate-controlled kinetic equation for a structure parameter was initially proposed by Goodeve and Whitfield [28]. Here, the structure parameter is controlled by the shear rate and used to adjust the viscosity of the material. De Souza Mendes and Thompson [29] find it more reasonable that structure evolution is controlled by the stress, since stress is needed to break bonds in the microstructure.

Another continuum approach to describe highly filled polymer liquids was described by Leonov [30]. This constitutive model also uses a kinetic approach for structure break-down and reformation. The rheological behavior is assumed to be dominated by filler–filler interaction. Simhambhatla and Leonov [31] extended this model to describe the rheological behavior of filled polymers with dominant filler–matrix interaction. They make use of a rate-controlled kinetic equation for a structure parameter and divide the stress into a contribution of the ‘free chains’ and one for the ‘trapped chains’. This model was validated with various rheological experiments by Joshi and Leonov [32].

Many phenomenological models that are based on the evolution of a structure parameter describe experimental data well. However, they provide no clear information about the microstructure of the material. Population Balance Models (PBMs) take into account the flow-induced changes of the microstructure and describe how these changes influence the bulk rheology of the material. In the last years, there has been a growing interest in developing thixotropic constitutive models from PBMs [33,34].

Recent work of Narayan and Palade compared a natural configuration approach and a structural parameter approach to model the thixotropic behavior in filled elastomers [35]. Although both approaches capture the rheological behavior reasonably well, they found there were still some aspects of the Payne effect that were inadequately described. Rendek and Lion [36] performed experiments to study the influence of strain-induced transient behavior of the Payne effect for filler-reinforced elastomers and also proposed a constitutive model. This model was later extended to investigate the influence of strain-amplitude and temperature [37]. Modeling the effects of strain-amplitude and frequency on the thixotropic behavior of carbon-black reinforced elastomers was done amongst others in [38,39].

In this paper, we will propose a straightforward experimental strategy to characterize the thixotropic behavior of reinforced rubber compounds. To this end, oscillatory measurements on a standard plate-plate rheometer are performed. Inspired by the Leonov modeling, the thixotropic behavior is modeled with a kinetic evolution equation for a structure parameter. Model predictions obtained with a rate- and stress-controlled kinetic equation are compared in order to investigate the typical features of these two approaches. The focus of this work is on this comparison and not the best quantitative description of experimental results, i.e., the modeling is more qualitative. The oscillatory shear measurements provide model parameters that can be used to predict steady shear results. What distinguishes our approach from previous work is the relatively straightforward coupling between the nonlinear viscoelastic behavior described by the constitutive model and the thixotropic behavior described by the structure parameter. To be able to predict properties of extrusion products of rubber compounds, numerical simulations can be a powerful tool [40,41,42,43]. For these simulations, the material behavior, including the rheological behavior, needs to be described. Although the presented model is a first-order model, only able to qualitatively predict the thixotropic behavior, the straightforward coupling between the nonlinear viscoelastic and thixotropic behavior maximizes its applicability and can help to give insight in the behavior under realistic processing conditions.

The paper is build up as follows: first the material and experimental strategy are introduced in Section 2. Section 3 introduces the rate- and stress-controlled thixotropic model. It is explained how the model parameters in both approaches are obtained. The results of the model predictions using both the rate- and stress-controlled equation are presented in Section 4, for oscillatory- and steady-shear. Finally, the results of both approaches are discussed in Section 5.

## 2. Materials and Methods

### 2.1. Materials

#### 2.1.1. Filled, Uncured Rubber Compound

Oscillatory-shear experiments are performed on a carbon-black filled styrene butadiene uncured rubber compound provided by Evonik Industries. The ingredients of the rubber compound are shown in Table 1 in parts per hundred rubber (phr).

#### 2.1.2. Sample Preparation

Plates are molded from the rubber compound by compressing the rubber with a hot press of Fontijne Holland (Type: TP 400) for three minutes, while keeping a constant normal force of 100kN applied to the rubber at 100°C. This is followed by compressing the rubber for three minutes at the same constant normal force at T=25°C. Teflon sheets are used to prevent the rubber from adhesing to the press plates. Circular samples with a diameter of 25mm and a thickness of 2mm are obtained by die-cutting them from the pressed plates. To reduce the torque needed to perform oscillatory-shear measurements at large strain amplitudes, also circular samples with a diameter of 8mm and a thickness of 2mm are used.

### 2.2. Methods

Rheological measurements on rubber compounds, i.e., thixotropic materials, are limited in terms of frequency in case of dynamic measurements, shear rate and temperature range. The latter due crosslinking at higher temperatures. Moreover, the performance and interpretation of dynamic measurements are more complicated for compounds than for regular polymers because of thixotropic behavior; properties can change during the measurements. The thixotropic behavior is caused by filler particles that create a reversible network, resulting in additional elastic behavior. This network can be (partly) destroyed by flow gradients and recovers after the flow has stopped. When the filler-network is completely destroyed one should observe the rheological characteristics of a filled polymer that is still a highly nonlinear viscoelastic material, i.e., very high zero-shear rate viscosity, clear shear thinning, normal stresses, glassy behavior at high frequencies. In this paper, we apply dynamic oscillatory shear measurements to characterize this behavior.

#### 2.2.1. Dynamic Oscillatory Shear Measurements

**Figure 2:**
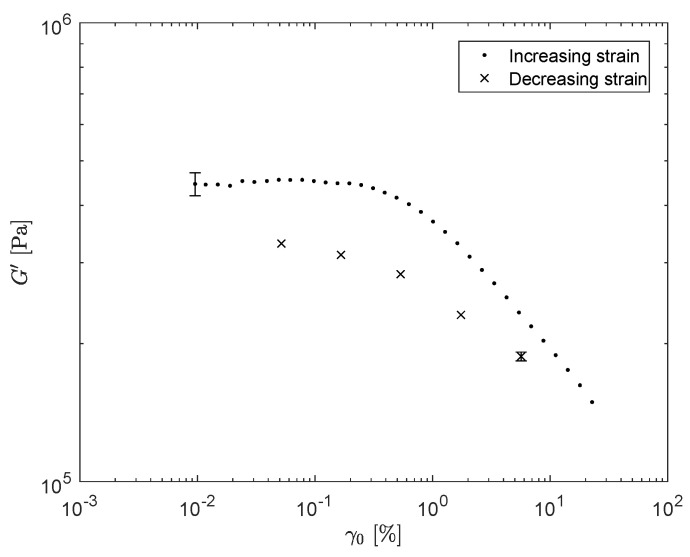
Strain sweep measurements of $G^{\prime }$ for increasing strain (black dots), followed by strain sweep measurements for decreasing strain (black crosses).

We will first focus on the results of dynamic measurements, i.e., oscillatory shear experiments, performed with a strain-controlled rheometer (TA-instruments, RDAIII), using a plate-plate geometry, from which a limited rheological spectrum is obtained. To determine the strain amplitude range for which the filler networks remain undamaged, a strain sweep is performed where the storage modulus $G^{\prime }$ is measured with increasing strain amplitude (T=75°C). To show that the decrease in $G^{\prime }$ is not caused by nonlinear viscoelasticity only, the measurement is immediately followed by a strain sweep with decreasing strain amplitude. The result is shown in Figure 2. This figure shows the effect of thixotropy, since the curve measured with decreasing strain amplitude shows lower values for $G^{\prime }$ as the curve measured with increasing strain amplitude. The measurements in this figure are repeated three times to show reproducibility.

**Figure 3:**
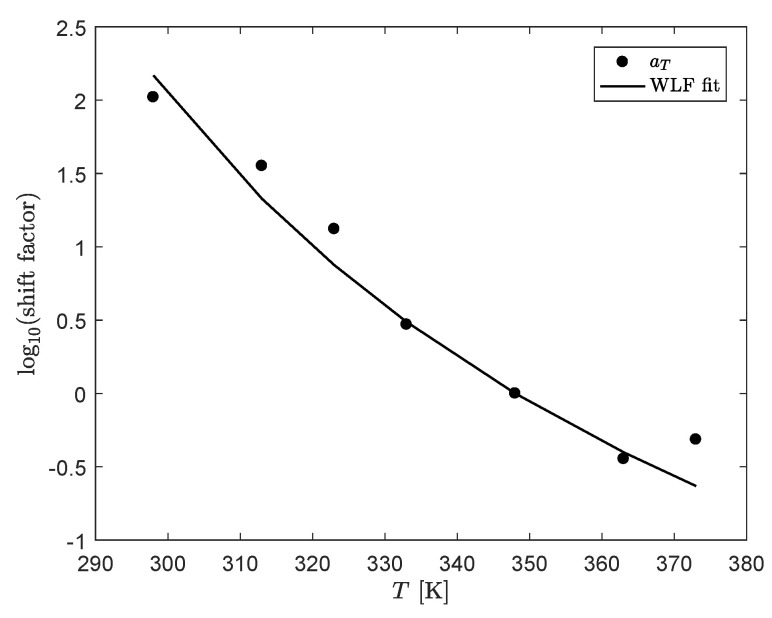
Horizontal $a_{T}$ shift-factors used in the time temperature superposition to construct mastercurves of $G^{\prime }$, $G^{\prime \prime }$ and δ and the obtained WLF-fit to the horizontal shift factors $a_{T}$.

To study the effect of deformation on the material properties of the compound, a relaxation spectrum of the undamaged material is needed to use as starting point. To this end, frequency sweeps are performed with a low strain amplitude of $\gamma_{0}=0.1\%$ at different temperatures. Time Temperature Superposition (TTS) is used to construct mastercurves for the storage- and loss-moduli ($G^{\prime }$ and $G^{\prime \prime }$), and the phase-angle δ, respectively. The horizontal shift-factors used in the TTS are shown in Figure 3. A vertical shift was not required. The WLF equation is used to fit the horizontal shift-factors $a_{T}$ [44]:

(1) $$\log_{10}\left(a_{T}\right)=\frac{-C_{1}\left(T-T_{\mathrm{ref}}\right)}{C_{2}+\left(T-T_{\mathrm{ref}}\right)},$$

where $C_{1}$ and $C_{2}$ are empirical constants obtained via a least-squares fit and their values can be found in Table 2 and $T_{\mathrm{ref}}=75\;{}^{\circ}C$ is the reference temperature of the mastercurves.

**Figure 4:**
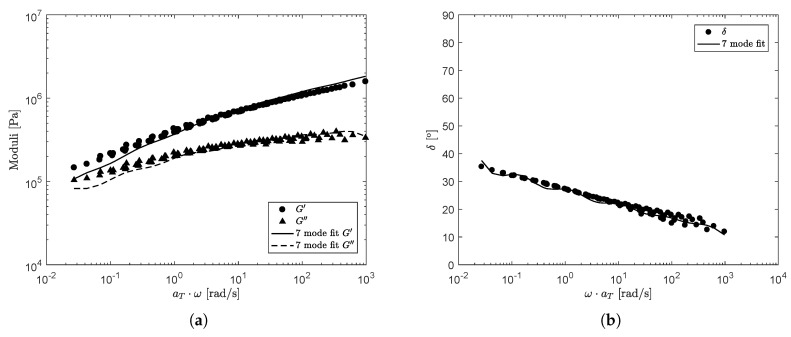
Mastercurves of the storage- and loss modulus measured with a strain amplitude of $\gamma_{0}=0.1\%$ at T=75°C (**a**) and the corresponding mastercurve of the phase-angle δ (**b**). Solid and dashed lines correspond to the 7-mode Maxwell fit of Table 3 to the data. The kinks in the fit of δ can be attributed to the contribution of the different modes, i.e., it is not a continuous spectrum. A smoother curve can be obtained by adding more modes in the suitable frequency range (λ=1/ω). However, this makes the modeling also more computationally expensive.

The constructed mastercurves for the storage- and loss-moduli and the phase-angle are shown in Figure 4. For the compound used in this paper, 7-modes seemed to be sufficient to obtain a good fit. For other compounds, the number of modes can be different. The relaxation spectrum for the undamaged material is obtained by performing a 7-mode least-squares fit on the mastercurves of $G^{\prime }$ and $G^{\prime \prime }$ and δ, using the Maxwell model [45]:

(2) $$G^{\prime }\left(\omega \right)=\sum_{k=1}^{m}\frac{G_{k}\lambda_{k}^{2}\omega^{2}}{1+{\left(\lambda_{k}\omega \right)}^{2}},\;G^{\prime \prime }\left(\omega \right)=\sum_{k=1}^{m}\frac{G_{k}\lambda_{k}\omega }{1+{\left(\lambda_{k}\omega \right)}^{2}},\;\delta \left(\omega \right)=\arctan\left(G^{\prime \prime }\left(\omega \right)/G^{\prime }\left(\omega \right)\right),$$

where *m* is the number of modes. The resulting fit is also shown in Figure 4. The values for the relaxation times $\lambda_{0,k}$ and moduli $G_{0,k}$ are shown in Table 3. Here, the subscript ${\left(\right)}_{0}$ indicates the spectrum used to characterize the undamaged material.

**Figure 5:**
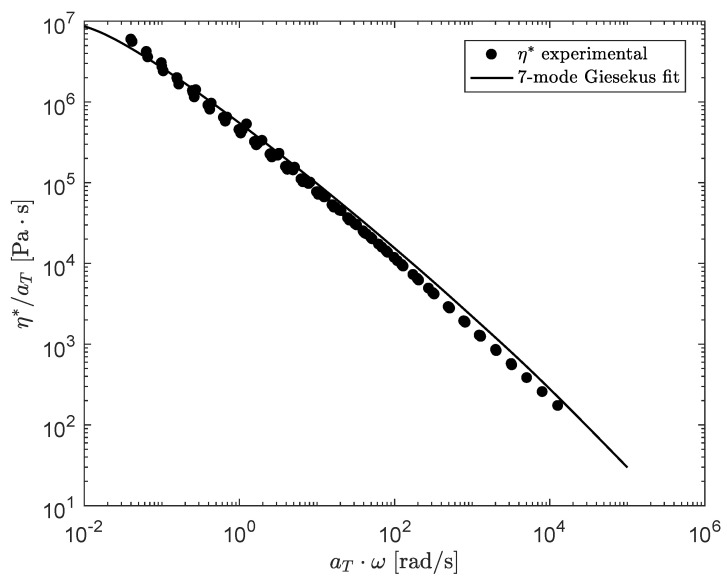
Fit of the Giesekus model to experimental data of the complex viscosity measured with oscillatory shear experiments.

To predict nonlinear viscoelastic behavior of the compounds, a Giesekus model is used to calculate the viscoelastic stress [46]:

(3) $$\lambda_{k}\left(\frac{D\tau_{k}}{Dt}-L\cdot \tau_{k}-\tau_{k}\cdot L^{T}\right)+\tau_{k}+\frac{\lambda_{k}\alpha_{k}}{\eta }\tau_{k}^{2}=2\eta_{k}D,\;\tau =\sum_{k=1}^{m}\tau_{k}$$

where D()/Dt denotes the material derivative, τ is the total viscoelastic stress tensor, L is the velocity gradient, $D=(\nabla u+\nabla u^{T})/2$ is the rate-of-deformation tensor, $\eta_{k}=G_{k}\lambda_{k}$ is the shear viscosity of mode *k* and $\alpha_{k}$ is the mobility parameter of mode *k* that influences shear-thinning. Note that it is not possible to perform steady shear measurements on the compounds without measuring the effect of thixotropy. To get an estimate of the mobility parameter $\alpha_{k}$ of the Giesekus model for every mode *k*, the model is fitted to the experimental data of the complex viscosity for the undamaged material. The result is shown in Figure 5. The nonlinear parameters obtained are given in Table 4.

**Figure 6:**
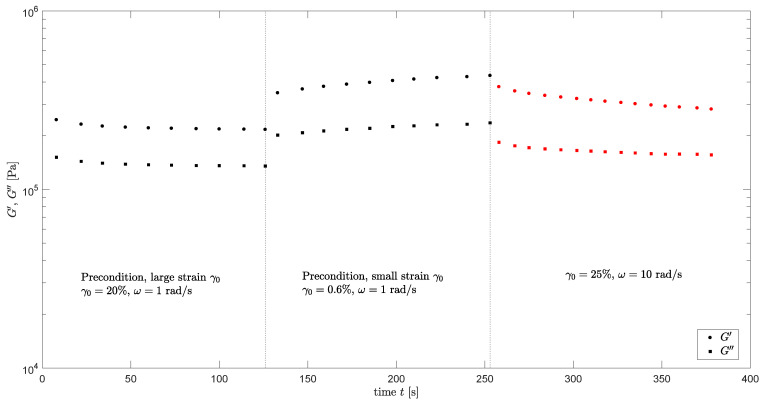
Measuring protocol to construct mastercurves of δ for different degrees of structural damage.

Next, mastercurves are constructed for different degrees of structural damage. A horizontal shift between mastercurves of the moduli of different degrees of structural damage could be observed for a large frequency range. Together with the assumption that structural damage leads to a ‘reduced’ time scale due to destruction of the elastic network in the material, this led to the choice to focus on the mastercurves of the phase-angle δ in this work. To erase any structural memory and make sure the starting properties of every sample are approximately the same, a pre-shear is applied. The measuring protocol of every point on the mastercurves with different degrees of structural damage in the material is shown in Figure 6 and is defined as follows:

1. For every sample, first a pre-shear (indicated in black) is applied by performing oscillatory shear measurements of t=120s, ω=1rad/s and a strain amplitude of $\gamma_{0}=20\%$ during which the structure will break down;
2. This is followed by a pre-shear during which the structure can (partially) recover again, by performing oscillatory shear measurements of t=120s, ω=1rad/s and a strain amplitude of $\gamma_{0}=0.6\%$;
3. These pre-shear measurements are followed by an oscillatory shear measurement of again t=120s, but for different frequencies ω and strain amplitudes $\gamma_{0}$ (indicated in red);
4. The storage and loss moduli at time t=350s are used to calculate the phase-angle δ and this gives one single point on the mastercurve with strain amplitude $\gamma_{0}$.

**Figure 7:**
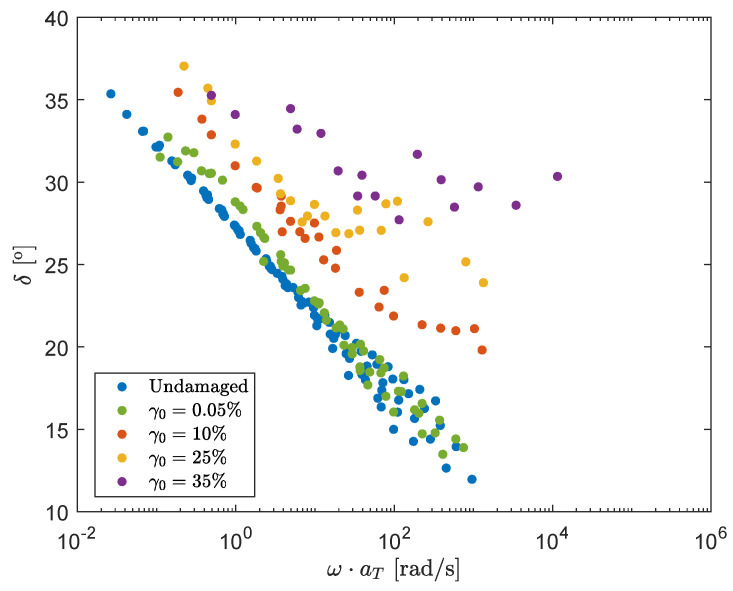
Mastercurves (T=75°C) of the phase-angle δ for different degrees of structural damage by applying different strain amplitudes $\gamma_{0}$.

The result for the mastercurves with different strain amplitudes, and therefore different degrees of structural damage, are shown in Figure 7. Here, every point is a separate experiment performed using the protocol as shown in Figure 6. It is important to note that the mastercurve for $\gamma_{0}=0.05\%$ is used to characterize the *recovery* behavior of the material. For this curve, only a pre-shear of t=120 s, ω=1 rad/s and a strain amplitude of $\gamma_{0}=20\%$ is applied, meaning that step 2 in the measuring protocol is skipped. This is followed by an oscillatory shear measurement of again t=120 s, but for different frequencies ω and a strain amplitude of $\gamma_{0}=0.05\%$ during which the material can recover.

#### 2.2.2. Steady Shear Measurements

Steady shear measurements are provided by VMI Holland B.V. and measurements were performed on an in-house made device. The compound is extruded in a closed, pressurized chamber containing a double-cone plate geometry. The pressure-method [47] is used to determine the shear viscosity η and the first normal stress difference coefficient $\Psi_{1}$ from the measurements. Measurements are performed for a temperature range *T* = 70–90 ${}^{\circ }$ C to construct mastercurves for T=75°C. Results are presented in Section 4.3.

## 3. Thixotropy Model

In this section, the assumptions and observations that lead to the model approach are presented. This is followed by the mathematical description of the rate- and stress-controlled thixotropy model used throughout this paper.

### 3.1. Model Definition

From Figure 7, the following assumptions/observations can be made:

- It is assumed that structural damage leads to a ‘reduced’ time scale due to destruction of the elastic network. Combined with the observation that structural damage causes the phase-angle δ to shift horizontally for a large range of frequencies, this leads to the assumption that the influence of structural damage on the phase-angle can be described with a horizontal shift factor $a_{\xi }$ for increasing the strain amplitude $\gamma_{0}$.
- For higher angular frequencies ω, there is an upswing in the phase-angle and the effect of damage can no longer be described by a pure horizontal shift.
- This upswing is more pronounced for higher levels of structural damage and starts at lower frequencies.

**Figure 8:**
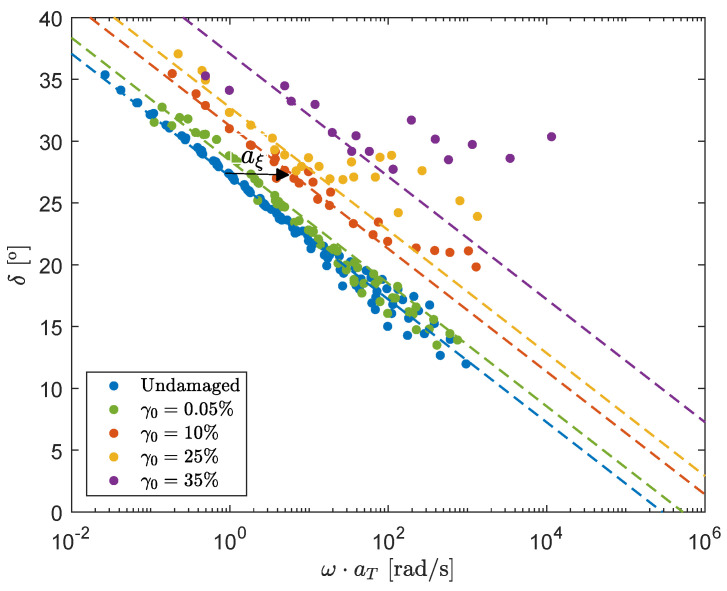
Mastercurves of different degrees of structural damage show a horizontal shift $a_{\xi }$ with respect to the undamaged mastercurve.

The undamaged mastercurve of δ shows a linear dependency on $\log_{10}\omega$. Therefore, a linear line can be fitted through this mastercurve. For small ω, a horizontal shift factor $a_{\xi }$ is applied to this linear fit, to obtain linear lines that fit the mastercurves for the different strain amplitudes $\gamma_{0}$. This is schematically depicted in Figure 8. This horizontal shift plays a crucial role in the model definition in this paper and the shift-factors $a_{\xi }$ for the different strain amplitudes can be found in Table 5.

Using a horizontal shift $a_{\xi }$ on the mastercurves of the phase-angle suggests that the effect of damage of the filler networks can be modeled by adjusting the relaxation times of the undamaged material. To show that this hypothesis is valid, the horizontal shift between the linear fits through the mastercurves are obtained and the relaxation times are adjusted accordingly:

(4) $$\lambda_{k}=\lambda_{0,k}\cdot \frac{1}{a_{\xi }}.$$

**Figure 9:**
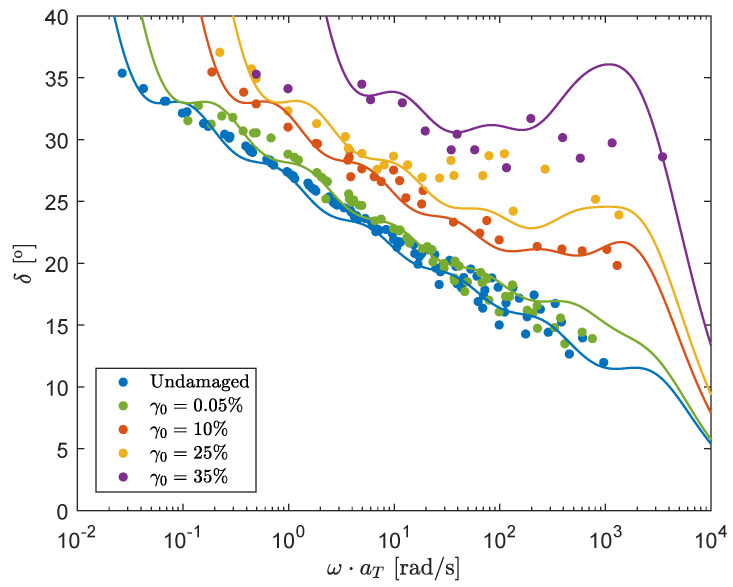
Results for the mastercurves of the phase-angle by adjusting the undamaged relaxation times with the horizontal shift-factors $a_{\xi }$.

For high frequencies, an upswing in the phase-angle is observed that becomes more pronounced for higher strain amplitudes. Structural damage is assumed to be dominant for the modes with the largest relaxation times, i.e., the relaxation times that are thought to be mostly related to the elastic filler network. Therefore, it is assumed that small relaxation times (corresponding to high frequencies) do not contribute to the elastic network. Following this reasoning, the horizontal shift of these modes should be limited. The measured upswing at high frequencies can now be used to determine this limited horizontal shift. Numerical experiments showed that the factor $1/a_{\xi }$ in Equation (4) for the two modes with the smallest relaxation times should be limited to describe the measured upswing at high frequencies. It was found that the experimental data were described best when the smallest relaxation time was not adjusted ($1/a_{\xi }=1$) and, for mode six, $1/a_{\xi }$ was limited to $\min(1/a_{\xi })=0.1$. The result is shown in Figure 9.

Even though upswings in δ have been measured in the past for elastic materials [48] and the upswing in the experimental data is captured well by limiting the damage of the smallest relaxation times, it has to be noted that the experimental results obtained at high frequencies are prone to experimental errors. Therefore, the measurements at high ω have to be considered less accurate than the measurements at small ω.

#### Extended Spectrum

So far, we have focused on the results of dynamic measurements, i.e., oscillatory shear experiments, from which a limited (7-mode) rheological spectrum is obtained. Based on a combination of arguments and some observations, this spectrum can be extended such that the shear rate range becomes applicable to realistic processing conditions.

**Figure 10:**
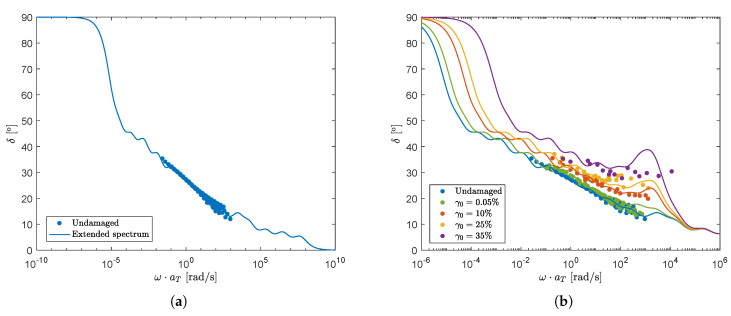
Phase-angle of the undamaged material as a function of frequency fitted with a 15-mode extended relaxation spectrum (**a**) and phase-angle as a function of frequency for different degrees of structural damage fitted with a 15-mode extended relaxation spectrum (**b**).

For a compound with network structures that are finite, i.e., without a network structure that spans the whole sample, the phase-angle has limits: (δ(ω→0)=90°), (δ(ω→∞)=0°). The relaxation spectrum can now be extended to lower and higher frequencies than the measurement range, such that the slope of the undamaged mastercurve is preserved over a large range, keeping the phase-angle limits in mind. The result is shown in Figure 10a. Notice that with this choice the material shows a Newtonian plateau for (very) small ω. When an ‘infinite network’ would exist in the sample, the phase-angle would show a limiting phase-angle close to zero for small ω, indicating that the material has a yield-stress. When choosing the right parameters, one can also model this behavior. However, we do not consider this possibility of yielding, since we have no experimental evidence for such behavior for the compound considered. The relaxation times $\lambda_{0,k}$ and moduli $G_{0,k}$ of the extended spectrum are given in Table 6.

Following the same reasoning as for the 7-mode spectrum, the horizontal shift for modes with small relaxation times, corresponding to high frequencies, is again limited. Using numerical experiments, the maximum adjustment factor for mode 10 was found to be $\min(1/a_{\xi })=0.1$. Modes 11–15 were not adjusted ($1/a_{\xi }=1$). The extended spectrum can now be used to fit the experimental mastercurves for the different degrees of structural damage. The result is shown in Figure 10b. Both the horizontal shift and the upswing are captured quite well. For practical reasons, we will not take into account the plateau limits in the phase-angle for the model predictions. Therefore, the 7-mode spectrum will be used in the remainder of this paper.

### 3.2. Mathematical Description

Results in the previous section show that adjusting the relaxation times of the undamaged spectrum with the horizontal shift $a_{\xi }$ between the different mastercurves of the phase-angle δ leads to a successful prediction of the measured behavior. Therefore, a structure parameter ξ is defined based on this horizontal shift-factor $a_{\xi }$ as $\xi =1/a_{\xi }$. If ξ=1, then the material is undamaged and the filler networks are intact, whereas $\xi =\xi_{\inf}$ indicates a completely damaged material where no network structures due to the fillers are left:

$$\xi =\left\{\begin{array}{cc} 1, & \;\mathrm{Undamaged};\;\mathrm{structures}\;\mathrm{intact}\\ \xi_{\inf}, & \;\mathrm{Maximum}\;\mathrm{damage}, \end{array}\right.$$

here, the minimum value for $\xi_{\inf}$ that can be used is $\xi_{\inf}=0$. The value for $\xi_{\inf}$ is related to the limited horizontal shift for small relaxation times introduced in the previous section to capture the upswing in δ for high frequencies. This means that for the 7-mode spectrum $\xi_{\inf}$ can be defined as follows:

(5) $$\xi_{\inf}=\left[0,\;0,\;0,\;0,\;0,\;0.1,\;1\right].$$

To model the thixotropic behavior, we compare a rate- and stress-controlled phenomenological structure parameter ξ to adjust the relaxation times of the undamaged spectrum, to obtain the relaxation spectrum of the damaged material. First, the mathematical description of both models is given, followed by the approach to obtain the different model parameters. The following kinetic equations are based on the work of Leonov et al. [30,31,32].

#### 3.2.1. Rate-Controlled Model

(6) $$\frac{D\xi }{Dt}=\frac{1-\xi }{\lambda_{\theta }}-E\gamma^{*}\left(\xi -\xi_{\inf}\right),$$

where D()/Dt=∂()/∂t+u·∇() is the material derivative, $\lambda_{\theta }$ is a characteristic time scale for the recovery of the material structure, $E=\sqrt{2\mathrm{tr}D^{2}}$ is a measure of the deformation rate based on the rate of deformation tensor D, corresponding to an effective shear rate in shear flows. Furthermore, $\gamma^{*}$ is a fitting parameter that indicates how much of the applied deformation leads to damage of the structure in the material and $\xi_{\inf}$ is a fitting parameter that limits the degree of damage that can be done to the material. This parameter is introduced, since the rheological characteristics of an unfilled polymer that is still a highly nonlinear viscoelastic material should be observed when the filler-network is completely destroyed.

For the oscillatory shear measurements, *E* is defined as follows:

(7) $$E\left(t\right)=|\gamma_{0}\omega \cos\left(\omega t\right)|,$$

which can be simplified to:

(8) $$\langle E\rangle =\frac{2\gamma_{0}\omega }{\pi },$$

to take *E* as the average over the period of oscillation [32].

#### 3.2.2. Stress-Controlled Model

(9) $$\frac{D\xi }{Dt}=\frac{1-\xi }{\lambda_{\theta }}-\frac{\tau_{c}\left(\xi \right)}{\eta_{0}}\tau^{*}\left(\xi -\xi_{\inf}\right),$$

where $\tau_{c}\left(\tau \right)$ is a characteristic stress in the material that is a function of the viscoelastic stress tensor τ, $\eta_{0}$ is the zero shear viscosity of the undamaged material and $\tau^{*}$ is a fitting parameter that describes how much of the present stress contributes to damage of the elastic network. Here, the equivalent von Mises shear stress is used as characteristic stress $\tau_{c}$ [49]:

(10) $$\tau_{c}=\sqrt{\frac{1}{2}\hat{\tau }:\hat{\tau }},$$

with $\hat{\tau }=\tau -\frac{1}{3}\left(\mathrm{tr}\;\tau \right)I$, the deviatoric part of the total viscoelastic stress tensor.

Since ξ is related to the horizontal shift between the mastercurves of δ, the relaxation times of the undamaged material $\lambda_{0,k}$ are now adjusted using the structure parameter ξ to obtain the relaxation times $\lambda_{k}$ of the damaged material:

(11) $$\lambda_{k}=\lambda_{0,k}\cdot \xi ,$$

where $\lambda_{0,k}$ are the initial relaxation times obtained form fitting the mastercurves of the undamaged material of Figure 4. Here, in contrast to the Leonov model, the coupling between the nonlinear viscoelastic behavior described by the constitutive equation and the structure parameter is very straightforward through the relaxation times.

### 3.3. Model Parameters

**Figure 11:**
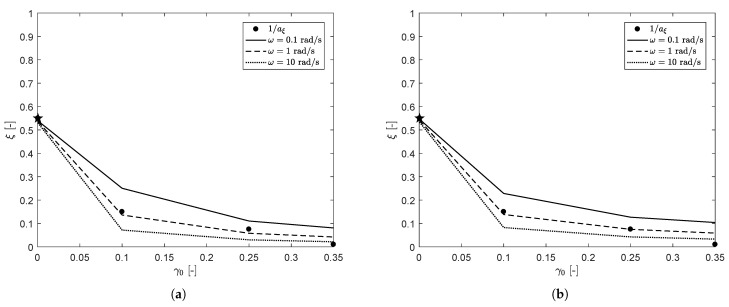
Structure parameter ξ obtained from the horizontal shift $a_{\xi }$ between the mastercurves of the phase-angle with different strain amplitudes fitted by the rate-controlled model (**a**) and the stress-controlled model (**b**) for different frequencies ω. The horizontal shift of the recovery curve $\gamma_{0}=0.05\;\%$ is indicated with a star.

We use one single structure parameter for all seven modes in the relaxation spectrum shown in Table 3 and therefore assume that ξ describes the overall structure in the material. The structure parameter for the different mastercurves in Figure 7 is obtained from the horizontal shift of the linear fits for the different strain amplitudes $\xi =1/a_{\xi }$ (see Table 5). This structure parameter is plotted as a function of the applied strain amplitude in Figure 11. Note that ξ for $\gamma_{0}=0.05\%$ is smaller than one, because this point indicates the horizontal shift of the recovery curve after the applied pre-shear of step 1 in the measuring protocol. Since this is the only ‘recovery’ curve, this point is indicated with a star. Since the horizontal shift is the same for a wide range of frequencies, the model parameters should be chosen such that the structure parameter ξ is approximately the same as this horizontal shift for different frequencies. Using the deformation history as shown in Figure 6, the evolution of the structure parameter ξ can be calculated using an explicit Euler method. It was found that for the rate-controlled model, the effect of ω was too pronounced if *E* was calculated using Equation (7). It can be argued that for high frequencies, a high strain-amplitude leads to a lower degree of damage than for low frequencies, because the structure acts more like an elastic solid at high ω. In order to capture this behavior, the effective shear rate *E* is given a power law-like dependence on the frequency ω:

(12) $$\langle E\rangle =\frac{2\gamma_{0}\omega^{p}}{{\left(\omega^{*}\right)}^{p-1}\pi }.$$

To avoid problems with the unit of $\gamma^{*}$, a characteristic $\omega^{*}$ is introduced. Here, $\omega^{*}=1$ rad/s since the horizontal shift $a_{\xi }$ is based on the shift at this frequency.

For the stress-controlled method, the characteristic stress is calculated as $\tau_{c}\left(\tau \right)=\left|G^{*}\right|\gamma_{0}$, where $G^{*}$ is the complex modulus $G^{*}=\sqrt{\left(G^{\prime 2}+G^{\prime \prime 2}\right)}$ calculated with the damaged spectrum, obtained from the previous time step. It is important to note that this stress only equals the von Mises shear stress if linear rheological behavior is assumed. Results of fitting the structure parameter as a function of strain amplitude for frequencies in the range ω=[0.1,1,10] rad/s for the two different models are shown in Figure 11. Here, it is tried to get the difference in the structure parameters for the different frequencies as small as possible, to obtain the same horizontal shift for a wide frequency range, while still using realistic values for the fitting parameters.

The obtained fitting parameters for both models are given in Table 7.

The values for $\xi_{\inf}$ were obtained in Section 3.1 from capturing the upswing while adjusting the relaxation times with the horizontal shift factor $\lambda_{0}=\lambda \cdot 1/a_{\xi }$.

## 4. Results

This section will show results of model predictions in oscillatory shear and steady shear for the rate- and stress-controlled model. First, dynamic oscillatory model predictions of the mastercurves of the phase angle are shown for both methods. This is followed by dynamic model predictions of oscillatory shear measurements of the damage recovery behavior for a fixed frequency of ω=1 rad/s and different strain amplitudes. Here, a distinction is made between predictions with a homogeneous flow assumption and more realistic non-homogeneous flow conditions where nonlinear viscoelasticity is taken into account. Finally, model predictions are shown for steady shear data for both approaches.

### 4.1. Mastercurve Model Predictions in Oscillatory Shear

**Figure 12:**
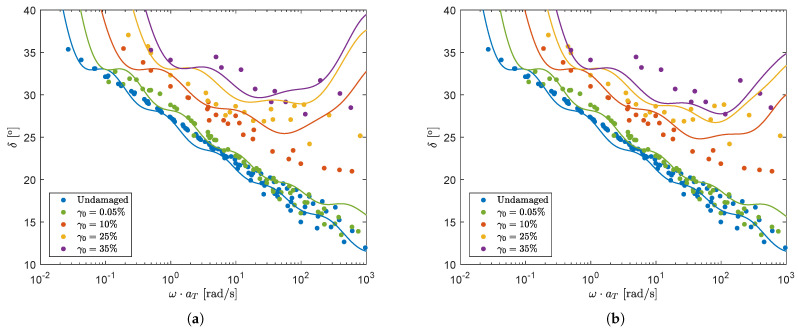
Model predictions of oscillatory shear measurements for the rate-controlled model, using a power law-like dependence on the frequency (**a**) and for the stress-controlled model assuming linear rheological behavior (**b**).

The 7-mode spectrum is used to calculate the model predictions in oscillatory shear. The structure parameter ξ is calculated for every point on the mastercurves of Figure 7, using an Explicit Euler method and taking the applied pre-shear into account. For the rate-controlled method, *E* is calculated using the power law-like dependence on ω as described in Equation (12). For the stress-controlled method, the characteristic stress is again calculated as $\tau_{c}\left(\tau \right)=\left|G^{*}\right|\gamma_{0}$, where $G^{*}$ is the complex modulus $G^{*}=\sqrt{\left(G^{\prime 2}+G^{\prime \prime 2}\right)}$ calculated with the damaged spectrum, obtained from the previous time step and, thus, linear rheological behavior is assumed. The results for the rate- and stress-controlled method are shown in Figure 12.

Results show that both models give a qualitative prediction of the measured mastercurves.

**Figure 13:**
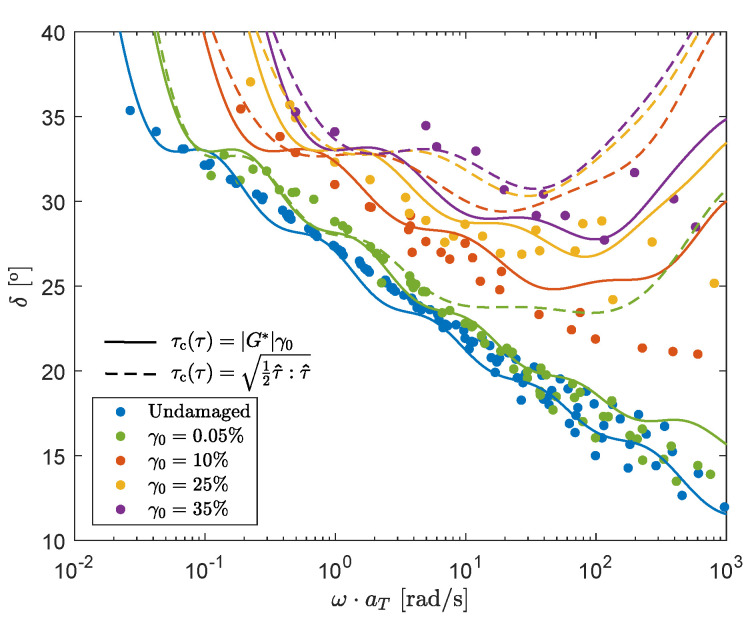
Model predictions of oscillatory shear measurements for the stress-controlled model, using $\tau_{c}\left(\tau \right)=\left|G^{*}\right|\gamma_{0}$ (solid lines) and $\tau_{c}=\sqrt{\frac{1}{2}\hat{\tau }:\hat{\tau }}$ (dashed lines).

The approximation of the characteristic stress $\tau_{c}\left(\tau \right)=\left|G^{*}\right|\gamma_{0}$ can only be used for small Weissenberg numbers. For large strain amplitudes and frequencies, normal stresses will start to play a role such that $|G^{*}|\gamma_{0}<\sqrt{\frac{1}{2}\hat{\tau }:\hat{\tau }}$. Figure 13 shows a comparison between the model predictions using $\tau_{c}\left(\tau \right)=\left|G^{*}\right|\gamma_{0}$ and the von Mises shear stress calculated using a Giesekus model.

This figure shows that for high strain amplitudes, but especially for high frequencies, assuming linear viscoelastic behavior is no longer allowed since model predictions using the von Mises shear stress are highly different from model predictions using $\tau_{c}\left(\tau \right)=\left|G^{*}\right|\gamma_{0}$. The von Mises stress is much higher than the shear stress for high frequencies, because normal stresses can no longer be neglected. Results show that, similar to the rate-controlled model, the frequency dependence is over-predicted compared to the experimental data. At these high frequencies, the measurements are also prone to experimental errors. The oscillatory shear measurements are therefore mainly used to obtain model parameters to do qualitative predictions for small ω and in steady shear. It should also be noted that in the fitting procedure linear viscoelastic behavior is assumed: $\tau_{c}\left(\tau \right)=\left|G^{*}\right|\gamma_{0}$. A more consistent fitting procedure, using the von Mises shear stress, might give better results for the oscillatory shear behavior. However, with the current model, this will most likely lead to an over-prediction of the rheological properties in steady shear.

### 4.2. Damage Recovery Behavior Model Prediction in Oscillatory Shear

The 7-mode relaxation spectrum in combination with the rate- and stress-controlled thixotropic models is used to model the dynamic behavior of the compound. The fitting parameters as found in Table 7 are used. Here, a distinction is made between a homogeneous flow assumption and a more realistic non-homogeneous flow as present in a plate-plate rheometer.

#### 4.2.1. Homogeneous Flow Assumption

**Figure 14:**
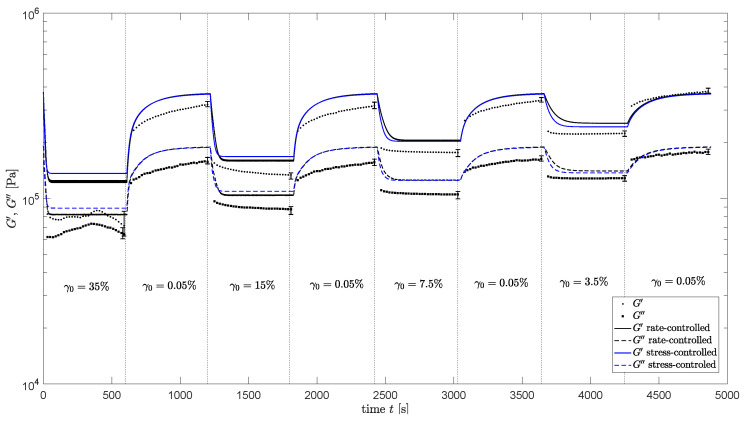
Dynamic experimental measurements of the damage–recovery behavior (ω=1 rad/s) of the compound (dots) and model predictions using a homogeneous flow assumption and the 7-mode relaxation spectrum (solid lines) for the rate-controlled method (black) and the stress-controlled method (blue).

Oscillatory shear measurements are performed for different strain amplitudes and ω=1 rad/s to find the damage–recovery behavior of the compound. A frequency of ω=1 rad/s is chosen to avoid experimental problems at high shear rates and problems with nonlinear rheological behavior. These measurements are again repeated three times to test the reproducibility. The evolution of the structure parameter ξ is again modeled using an explicit Euler scheme to solve Equations (6) and (9), using *E* as defined by Equation (12) for the rate-controlled method, and the stress $\tau_{c}\left(\tau \right)=\left|G^{*}\right|\gamma_{0}$ calculated with ξ from the previous time step for the stress-controlled method. The flow is assumed to be homogeneous (no radial dependence) and nonlinear viscoelasticity is neglected. The undamaged relaxation times as listed in Table 3 are adjusted using Equation (11), and the storage and loss moduli are calculated using Equation (2). The experimental results and the model prediction for the storage- and loss moduli are shown in Figure 14 for both methods. This figure shows that the damage–recovery behavior of the compound is captured quite well for both approaches.

#### 4.2.2. Non-Homogeneous Flow

In the previous section, the assumption of homogeneous flow was used. However, in reality, the flow in a plate-plate rheometer is non-homogeneous and, thus, the shear rate is linearly dependent on the radius *r* in the sample. This means the structure parameter ξ and the material properties will also be *r*-dependent. Therefore, full computations are performed where a sinusoidal strain is applied that depends on the radial position *r* in the sample:

(13) $$\gamma \left(r,t\right)=\frac{r}{R_{\mathrm{plate}}}\cdot \gamma_{0}\sin\left(\omega t\right),$$

(14) $$E\left(r,t\right)=\frac{r}{R_{\mathrm{plate}}}\cdot \gamma_{0}\omega \cos\left(\omega t\right),$$

where *r* is the radial position in the sample and $R_{\mathrm{plate}}$ is the radius of the plate of the rheometer. Nonlinear viscoelastic behavior is taken into account by using a Giesekus model to calculate the visoelastic stresses. An Explicit Euler scheme is used to calculate the viscoelastic stress from Equation (3) as a function of time and radius. From this stress, the torque can be calculated [50]:

(15) $$M=2\pi \int_{0}^{R}r\tau_{\theta z}\left(r\right)rdr.$$

The rheometer software determines the stress $\tau_{\theta z}$ from this torque with the following equation:

(16) $$\tau_{\theta z}=\frac{2M}{\pi R^{3}}.$$

For viscoelastic fluids, this equation is a linear approximation. From this stress, $G^{\prime }$ and $G^{\prime \prime }$ can be calculated. The viscoelastic stress is calculated using the Giesekus model with $\alpha_{k}$ parameters as can be found in Table 4.

**Figure 15:**
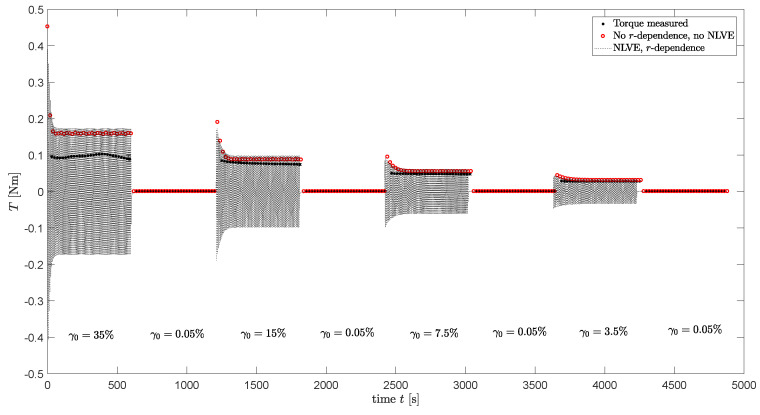
Dynamic experimental measurements of the torque during the damage–recovery behavior measurements of the compound (dots), model predictions using a homogeneous flow assumption and the 7-mode relaxation spectrum (blue dots) and model predictions taking *r*-dependence and Nonlinear Viscoelastic (NLVE) behavior into account (dotted line) for the rate-controlled model.

**Figure 16:**
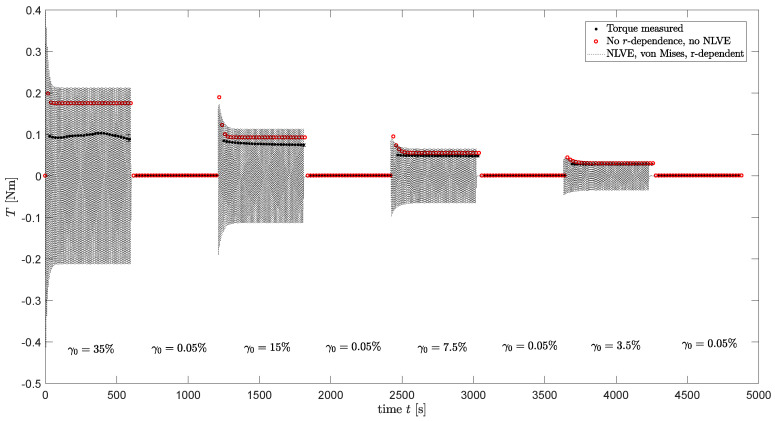
Dynamic experimental measurements of the torque during the damage–recovery behavior measurements of the compound (dots), model predictions using a homogeneous flow assumption and the 7-mode relaxation spectrum (blue dots) and model predictions taking *r*-dependence and nonlinear viscoelastic behavior into account (dotted line) for the stress-controlled model.

The evolution of the structure parameter is also calculated using an explicit Euler method, taking the radial dependence of the applied strain into account. The relaxation times are adjusted using the calculated structure parameter and used in the calculations of the viscoelastic stress tensor. For the stress-controlled method, ξ is calculated using the von Mises shear stress as characteristic stress $\tau_{c}$. To this end, the von Mises stress calculated from τ in the previous time step is used. The torque measured during the oscillatory shear measurements of the damage–recovery behavior of Figure 14 is now calculated using Equation (15). Using this method, both the non-homogeneous flow and nonlinear viscoelasticity are taken into account. The result is shown in Figure 15 for the rate-controlled method and, in Figure 16, for the stress-controlled method. These figures show that for ω=1 rad/s and the strain amplitudes applied in the oscillatory shear experiments, neglecting nonlinear viscoelastic behavior and radial dependence of the strain still gives qualitative results.

### 4.3. Model Prediction in Steady Shear

Finally, model predictions using the rate- and stress-controlled method in steady shear are presented. To this end, an equilibrium value of the structure parameter is used to calculate the viscosity η and the first normal stress difference coefficient $\Psi_{1}$ for different shear rates. The equilibrium value of the structure parameter $\xi_{\mathrm{eq}}$ can be calculated by setting the time derivative in Equations (6) and (9) equal to zero. This gives the following expression for $\xi_{\mathrm{eq}}$, for the rate- and stress-controlled method, respectively:

(17) $$\xi_{\mathrm{eq}}=\frac{1+E\gamma^{*}\xi_{\inf}\lambda_{\theta }}{1+E\gamma^{*}\lambda_{\theta }},$$

(18) $$\xi_{\mathrm{eq}}=\frac{1+\frac{\tau_{c}\left(\tau_{\mathrm{eq}}\right)}{\eta_{0}}\tau^{*}\xi_{\inf}\lambda_{\theta }}{1+\frac{\tau_{c}\left(\tau_{\mathrm{eq}}\right)}{\eta_{0}}\tau^{*}\lambda_{\theta }}.$$

For the stress-controlled equation, the von Mises stress $\tau_{c}\left(\tau \right)$ is calculated using a Giesekus model. Here, Newton–Raphson iteration is used to obtain the steady state viscoelastic stress tensor for a specific shear rate. Picard iteration is performed to obtain $\tau_{c}\left(\tau_{\mathrm{eq}}\right)$. By calculating the equilibrium value of the structure parameter for different shear rates, the 7-mode relaxation spectrum of the undamaged material is adjusted and the viscosity η and $\Psi_{1}$ can be calculated using the analytical solutions of the Giesekus model:

(19) $$\eta \left(\dot{\gamma }\right)=\sum_{k=1}^{m}\frac{\eta_{k}{\left(1-f_{k}\right)}^{2}}{1+\left(1-2\alpha_{k}\right)f_{k}},$$

(20) $$\Psi_{1}\left(\dot{\gamma }\right)=\sum_{k=1}^{m}\frac{2\eta_{k}\lambda_{k}f_{k}\left(1-\alpha_{k}f_{k}\right)}{\alpha_{k}\left(1-f_{k}\right){\left(\lambda_{k}\dot{\gamma }\right)}^{2}},$$

where $f_{k}$ is expressed as follows:

(21) $$f_{k}=\frac{1-\chi_{k}}{1+\left(1-2\alpha_{k}\right)\chi_{k}},$$

with,

(22) χk=[1+16αk(1−αk)(λkγ˙)2]12−18αk(1−αk)(λkγ˙)212.

**Figure 17:**
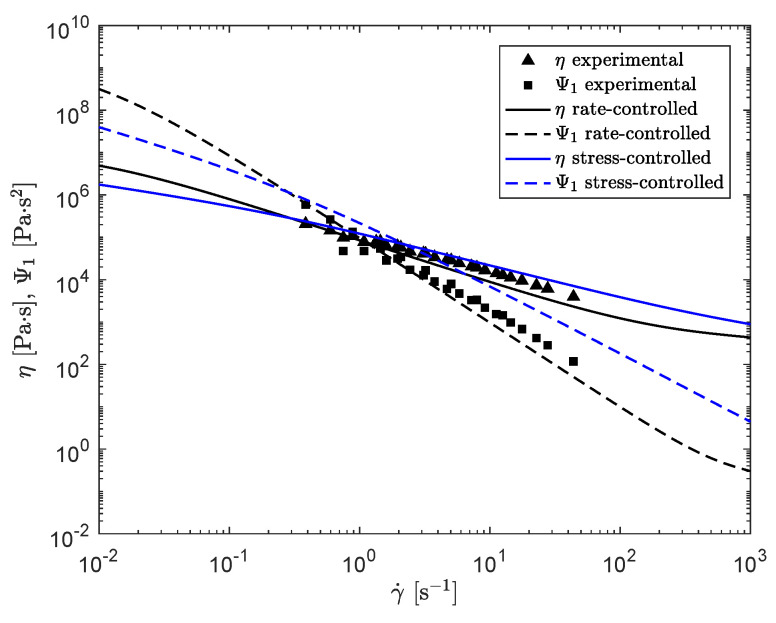
Steady shear predictions of the viscosity η and first normal stress difference coefficient $\Psi_{1}$ for the rate-controlled (black) and stress-controlled (blue) method and experimental data provided by VMI Holland B.V. (triangles and squares).

The result is shown in Figure 17. Here, the black lines indicate the result for the rate-controlled model, whereas the blue lines present the result for the stress-controlled model.

**Figure 18:**
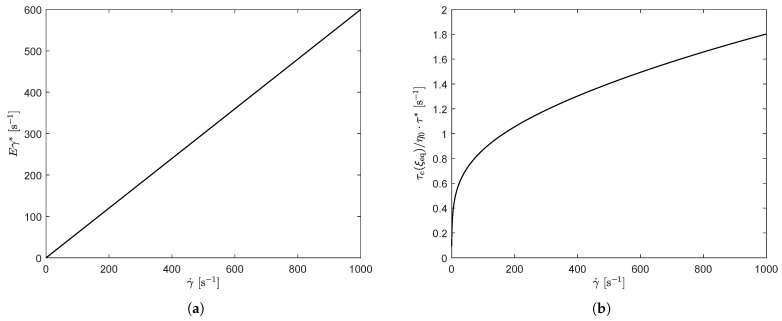
Model predictions of the damage term $E\gamma^{*}$ as a function of shear rate for the rate-controlled model (**a**) and $\tau_{c}\left(\tau \mathrm{eq}\right)/\eta_{0}\cdot \tau^{*}$ for the stress-controlled model (**b**).

This figure shows that both, the viscosity and the first normal stress difference coefficient are predicted to be lower for small shear rates and higher for large shear rates for the stress-controlled model compared to the rate-controlled model. Both models capture the experimental trends reasonably well, but the prediction of $\Psi_{1}$ is too high for larger shear rates, for the stress-controlled approach. Figure 18 shows the damage terms of both approaches as a function of shear rate. This figure shows that for the rate-controlled model, the damage term is linearly dependent on shear rate, whereas for the stress-controlled model, this dependency is no longer linear.

**Figure 19:**
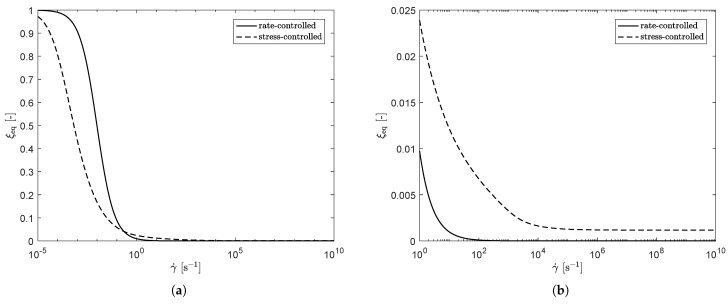
Model predictions of $\xi_{\mathrm{eq}}$ as a function of shear rate for both approaches (**a**) and a zoom-in at high shear rates (**b**).

Figure 19a shows $\xi_{\mathrm{eq}}$ as a function of shear rate for both approaches. This figure shows a clear difference between the rate- and stress-controlled approach, leading to the differences in steady-shear predictions. Figure 19b shows a zoomed-in version of (a). Here, it is observed that for the stress-controlled approach, $\xi_{\mathrm{eq}}$ reaches a plateau at $\xi_{\mathrm{eq}}\neq 0$ for high shear rates, whereas it goes to zero for the rate-controlled approach. This means that the stress-controlled approach always predicts a higher level of structure present in the material at high shear rates, compared to the rate-controlled approach, leading to larger relaxation times and, therefore, a higher viscosity and first normal stress difference coefficient.

**Figure 20:**
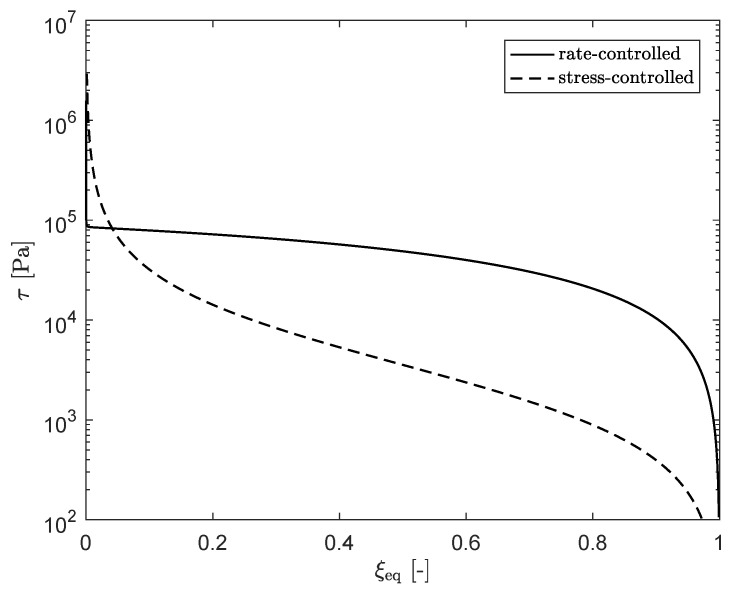
Shear-stress τ as a function of $\xi_{\mathrm{eq}}$ for the rate- and stress-controlled approach.

Figure 20 shows the shear stress τ as a function of $\xi_{\mathrm{eq}}$ for both approaches. Compared to the rate-controlled approach, the stress for the stress-controlled approach is always lower, except for very low $\xi_{\mathrm{eq}}$, where the plateau in $\xi_{\mathrm{eq}}$ is reached.

#### Transient Shear Rheology

**Figure 21:**
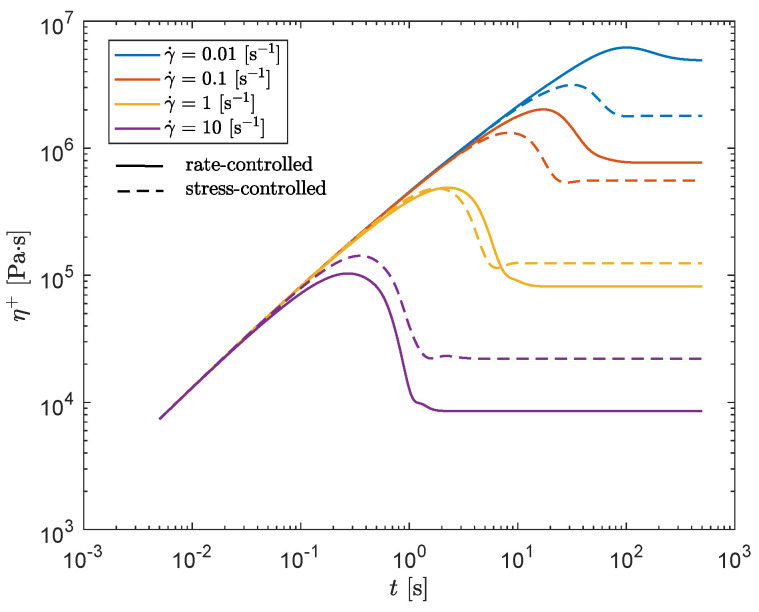
Transient viscosity as a function of time for different shear rates for the rate-controlled (solid lines) and stress-controlled model (dashed lines).

The transient viscosity can also be calculated for both models and different shear rates. The result is shown in Figure 21.

This figure also shows the effect of the damage-dependent characteristic stress in the stress-controlled approach. For smaller shear rates, the stress-controlled method predicts a smaller viscosity compared to the rate-controlled method. This agrees with the smaller equilibrium structure parameter for small shear rates, as was shown in Figure 20. The viscosity also reaches its equilibrium value faster for the stress-controlled method at small shear rates. For high shear rates, however, an opposite effect is observed. This is most likely caused by the damage-dependent characteristic stress in the evolution equation for the structure parameter. At large shear rates, the degree of damage is more severe, which reduces ξ and thus the relaxation times. This reduces the characteristic stress in the material, which in turn reduces ξ.

## 5. Discussion and Conclusions

In this paper, we presented an experimental strategy to characterize the rheological behavior of filled, uncured rubber compounds. It is tried to keep the approach as simple as possible to maximize applicability and keep the characterization straightforward. To this end, oscillatory shear experiments on a regular plate-plate rheometer were performed.

Measurements were performed for different strain amplitudes, to create mastercurves of the phase-angle for different degrees of structural damage in the material. It was found that the network destruction causes a horizontal shift $a_{\xi }$ in the mastercurves of δ. This shift is approximately the same for a large range of frequencies and used to construct a structure parameter $\xi =1/a_{\xi }$. This structure parameter is used to define the evolution of the degree of structural damage in the material and to adjust the relaxation times of the undamaged material $\lambda_{0}$ accordingly.

A rate- and stress-controlled kinetic equation for the evolution of the structure parameter is compared to qualitatively describe the thixotropic behavior in the material. The evolution of the structure parameter can be modeled in time, using an explicit Euler method. The model parameters are obtained by a least-square fitting approach based on the oscillatory shear measurements. Results show that the frequency dependence is not captured well by neither the rate- nor the stress-controlled model without additional model modifications. At these high frequencies, the oscillatory shear measurements are also prone to experimental errors. It therefore has to be concluded that, although the oscillatory shear measurements are used to obtain the model parameters for the kinetic structure parameter equations, we run into limitations of both, the measurements and the models, when normal stresses become significantly large.

The experimental damage–recovery behavior measured in oscillatory shear can be described reasonably well with both approaches for ω=1 rad/s. For the strain amplitudes used in the oscillatory shear measurements in this work and ω=1 rad/s, it is shown that it is allowed to neglect nonlinear viscoelasticity, and non-homogeneous flow in the plate-plate rheometer to model the damage–recovery experiments. However, this can be taken into account when larger strain amplitudes or frequencies are desired.

The model parameters obtained from the oscillatory shear measurements are used to perform steady shear model predictions. The results show that, using the model parameters obtained from the oscillatory measurements, the measured trends are qualitatively captured by both models. It was found that the stress-controlled approach under-predicts the degree of damage for large deformations. Perhaps, the approach to obtain the model parameters can be adjusted to obtain a better fit in steady shear for the stress-controlled model. More research is needed to find a more suitable approach.

Rubber compounds are complex materials containing many additives. To unravel the thixotropic behavior and do quantitative predictions, a more detailed model should be developed and the influence of these additives needs to be studied systematically. The frequency and temperature dependency of the filler-filler or filler-polymer networks has to be studied in future work. We believe that the thixotropy model and experimental strategy presented in this paper can be a starting point for characterizing the thixotropic behavior of such compounds. The trends measured in steady shear, using model parameters obtained from oscillatory shear measurements, are captured reasonably well for both models. As such, a qualitative prediction of the rheological behavior under relevant processing conditions can be done. However, more research is needed to systematically obtain the fitting parameters and give more quantitative results in both oscillatory and steady shear.

## Figures and Tables

**Table 1:** Ingredients of the rubber compound in phr.

	Ingredient	[ phr]
**Stage 1**	BUNA VSL 4526-2	96.25
	CB1220	30
	N339	72
	ZnO RS	2.00
	Stearic acid	1.00
	Vivatec 500	8.75
	Vulkanox HS/LG	1.50
	Vulkanox 4020/LG	2.00
	Protektor G 3108	2.00
**Stage 2**	Vulkacit CZ/EG C	1.60
	Sulfur	1.40

**Table 2:** WLF parameters.

$C_{1}$ [-]	$C_{2}$ [K]
4.54	154.64

**Table 3:** Relaxation spectrum of a 7-mode Maxwell fit of $G^{\prime }$ and $G^{\prime \prime }$ of the undamaged material.

Mode	$G_{0,k}$ [Pa]	$\lambda_{0,k}$ [s]
1	$1.25\times 10^{5}$	75.93
2	$1.45\times 10^{5}$	6.16
3	$2.27\times 10^{5}$	0.91
4	$3.16\times 10^{5}$	0.14
5	$4.05\times 10^{5}$	0.022
6	$5.30\times 10^{5}$	0.0032
7	$6.80\times 10^{5}$	0.0003

**Table 4:** Nonlinear parameter $\alpha_{k}$ used in the Giesekus model for nonlinear viscoelastic stress calculations.

Mode	$\alpha_{k}$ [-]
1	0.4
2	0.4
3	0.4
4	0.4
5	0.4
6	0.4
7	0.25

**Table 5:** Horizontal shift $a_{\xi }$ for the different strain amplitudes in Figure 7.

$\gamma_{0}$ [%]	$a_{\xi }$ [-]	$1/a_{\xi }$ [-]
0.05	1.81	0.55
10	6.67	0.15
25	13.33	0.075
35	100	0.01

**Table 6:** Extended 15-mode relaxation spectrum for the undamaged material.

Mode	$G_{0,k}$ [Pa]	$\lambda_{0,k}$ [s]
1	$0.02\times 10^{5}$	$1\times 10^{5}$
2	$0.04\times 10^{5}$	$2\times 10^{4}$
3	$0.1\times 10^{5}$	$3\times 10^{3}$
4	$0.3\times 10^{5}$	$4\times 10^{2}$
5	$0.7\times 10^{5}$	50.00
6	$1.3\times 10^{5}$	5.80
7	$2.1\times 10^{5}$	0.91
8	$3.0\times 10^{5}$	0.14
9	$4.0\times 10^{5}$	0.022
10	$5.0\times 10^{5}$	0.0032
11	$6.80\times 10^{5}$	0.0003
12	$7.00\times 10^{5}$	$50\times 10^{-6}$
13	$7.00\times 10^{5}$	$40\times 10^{-7}$
14	$8.00\times 10^{5}$	$30\times 10^{-8}$
15	$8.00\times 10^{5}$	$20\times 10^{-9}$

**Table 7:** Fit parameters used to fit ξ as a function of the strain amplitude $\gamma_{0}$.

$\lambda_{\theta }$ [s]	$\gamma^{*}$ [-]	*p* [-]	$\tau^{*}$ [-]
170	0.6	0.3	17.6

## Data Availability

The data that support the findings of this study are available from the corresponding author upon reasonable request.
